# Intra-articular corticosteroid knee injection induces a reduction in meniscal thickness with no treatment effect on cartilage volume: a case–control study

**DOI:** 10.1038/s41598-020-70064-4

**Published:** 2020-08-14

**Authors:** Jean-Pierre Pelletier, Jean-Pierre Raynauld, François Abram, Marc Dorais, Patrice Paiement, Johanne Martel-Pelletier

**Affiliations:** 1 Osteoarthritis Research Unit, University of Montreal Hospital Research Centre (CRCHUM), 900 Saint-Denis, Suite R11.412A, Montreal, QC H2X 0A9 Canada; 2 Medical Imaging Research and Development, ArthroLab Inc., Montreal, QC H2K 1B6 Canada; 3 StatSciences Inc., Notre-Dame-de-L’Île-Perrot, QC J7V 0S2 Canada; 4 ArthroLab Inc., Montreal, QC H2K 1B6 Canada

**Keywords:** Rheumatology, Musculoskeletal system, Rheumatic diseases, Medical research, Outcomes research, Translational research

## Abstract

Although intra-articular corticosteroid injections (IACI) are commonly used for the treatment of knee osteoarthritis (OA), there is controversy regarding possible deleterious effects on joint structure. In this line, this study investigates the effects of IACI on the evolution of knee OA structural changes and pain. Participants for this nested case–control study were from the Osteoarthritis Initiative. Knees of participants who had received an IACI and had magnetic resonance images (MRI) were named cases (n = 93), and each matched with one control (n = 93). Features assessed at the yearly visits and their changes within the follow-up period were from MRI (cartilage volume, meniscal thickness, bone marrow lesions, bone curvature, and synovial effusion size), X-ray (joint space width), and clinical (Western Ontario and McMaster Universities Osteoarthritis Index [WOMAC] pain score) data. Participants who received IACI experienced a transient and significantly greater rate of loss of the meniscal thickness (*p* = 0.006) and joint space width (*p* = 0.011) in the knee medial compartment in the year they received the injection, compared to controls. No significant effect of the IACI was found on the rate of cartilage loss nor on any other knee structural changes or WOMAC pain post-treatment. In conclusion, a single IACI in knee OA was shown to be safe with no negative impact on structural changes, but there was a transient meniscal thickness reduction, a phenomenon for which the clinical relevance is at present unknown.

## Introduction

Osteoarthritis (OA) is one of the most frequent chronic pain conditions for which the treatment remains largely symptomatic^1–7^. Oral treatments with analgesics, nonsteroidal anti-inflammatory drugs (NSAIDs), COX-2-selective inhibitors, and symptomatic slow-acting drugs (SYSADOAs) are the most common agents used in clinical practice^2^. In addition, local treatments with injectable hyaluronic acid (HA) or corticosteroids (IACI) have also been used successfully for many years^8,9^.

Although several studies have explored the efficacy of IACI to treat knee OA symptoms and the extent and length of response to a single treatment, meta-analysis indicated that the clinical benefit of an IACI after one to six weeks was unclear^8^. Recent clinical trials, however, suggested that repeated treatment with an extended-release formulation of triamcinolone acetonide significantly prolongs and amplifies the symptomatic benefit with no deleterious effect on joint structure when assessed by X-rays^10,11^. In a recent review^12^, it was however, suggested that this particular issue about the safety of IACI remains to be determined until examined more closely.

In the context of OA disease modifying effects of the steroid treatments, only two clinical trials explore the effects of repeated injections of IACI^13,14^. A first clinical trial^13^, in which repeated injections of therapeutic dosage of triamcinolone acetonide were given every three months for two years, showed an improvement in disease symptoms (pain, stiffness) over placebo (saline), with no effect on the progression of knee OA assessed by X-ray. A second clinical trial^14^, with a similar design and assessments performed using magnetic resonance imaging (MRI), found in the index compartment of the triamcinolone acetonide treatment group a small but significant reduction in cartilage volume using a quantitative measure, however, this finding was not corroborated with a semi-quantitative method. Moreover, the triamcinolone injections had no effect on the level of pain. The authors^14^ concluded that the findings raised the possibility for long-term adverse consequences on the health of the joint.

The Osteoarthritis Initiative (OAI) cohort presents an exceptional opportunity to study knee OA patients in their natural environment over an extended period of time, as well as the efficacy of some therapeutic interventions in real-life scenarios. Studies that used the OAI cohort have identified the determinants predicting the response to intra-articular hyaluronic acid (HA) treatment in symptomatic knee OA, as well as the effects of treatment such as SYSADOAs and other drugs used for the symptomatic treatment of OA on the evolution of joint structural changes and disease outcome^15–21^.

The main aim of this nested, case-controlled study was to identify and compare, for the first time using OAI participants, the impact of IACI on knee structural changes including various knee articular tissues. Participants received one treatment with IACI in one or both knees and MRI exams were available surrounding the yearly follow-up periods before, during and after the treatment. Clinical (symptoms) and X-ray (joint space width [JSW]) data were also analyzed.

## Methods

### Study patients

**Figure 1:**
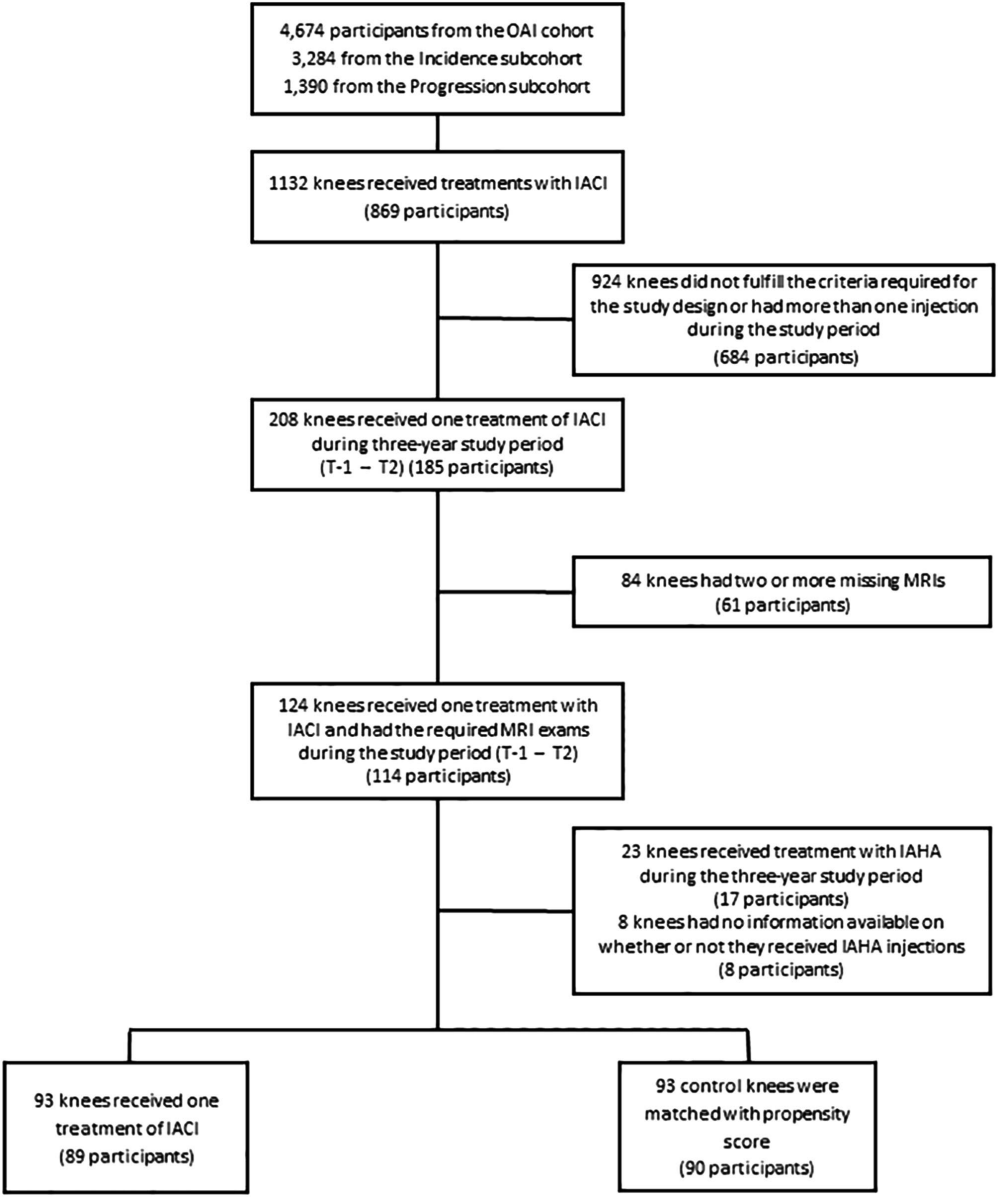
Participant disposition. Knees in intra-articular corticosteroid injection (IACI) group received treatment between T0 and T1. T-1, Baseline; T0, month 12; T1, month 24; T2, month 36; T3, month 48. IAHA, Intra-articular Hyaluronic Acid Injection; MRI, Magnetic Resonance Imaging; OAI, Osteoarthritis Initiative.

The OAI cohort used in this study complies with the Declaration of Helsinki. Written informed consent was obtained from each participating subject and the study was approved by the Institutional Review Board (IRB) of the OAI Coordinating Center at the University of California, San Francisco and the IRBs of each site. Data were from participants from the Incidence and Progression subcohorts from the OAI database, which is publicly available (https://nda.nih.gov/oai) and the participant disposition is in Fig. 1. The selection of subjects was based on the following question asked at each visit: "During the past 6 months, have you had a treatment with injections of steroids (cortisone, corticosteroids) in either of your knee(s) (right, left, or both) for your arthritis?" Selected participants were those who had received one treatment with IACI in one or both knees during one or more of the follow-up periods (Supplementary Fig. S1). Moreover, participants should not have received an IACI before the T-1 (baseline) visit or during the follow-up periods preceding (T-1–T0) or following (T1–T2; T2–T3) the injection in which T-1 indicates baseline; T0, month 12; T1, month 24; T2, month 36; and T3, month 48. In addition, to be included and to allow for the comparison of the structural effects of the treatment, MR images had to be available on at least three of the four yearly visits that took place before, during, and after the IACI. Of note, as in the OAI database, there are no MR images available for the 60 months (M) and 84 M visits. Missing MRI exam at T2 was allowed, however when possible, the T3 exam was used instead (20 knees, 19 patients), and the yearly mean structural changes over T1–T3 were assessed and used as a replacement.

Using a propensity score method^22^, one control was matched to each case (IACI group). The propensity score was based on various covariates measured at the last visit prior to the injection T0, which included age, gender, pain or arthritis medication, body mass index (BMI), JSW, cartilage volume in the medial and lateral compartments, bone marrow lesion (BML), Western Ontario and McMaster Universities Osteoarthritis Index (WOMAC) pain, and for meniscal extrusion, height, which was assessed at baseline (T-1). Data collected for study participants included demographics, WOMAC pain, Kellgren and Lawrence (KL) grades, and JSW on each of the yearly visits, when available.

### Demographics, clinical and imaging characteristics

Data on OA patient demographics, WOMAC pain, imaging (KL, JSW, MRI), and concomitant arthritis drug treatments were obtained from the OAI (https://data-archive.nimh.nih.gov/oai/). The values of the medial compartment JSW were from the central reading. The MR images were assessed by ArthroLab Inc. (Montreal, Quebec, Canada).

Knee MRI acquisitions were acquired using a double-echo steady-state (DESS) imaging protocol from 3.0 T apparatus (Magnetom Trio, Siemens, Germany) at the four OAI clinical centers as per OAI protocol. Fully automated and validated quantitative MRI technology was used to assess the cartilage volume^23^, bone curvature^18^, bone marrow lesion^24^, synovial fluid effusion size^25^, and a validated scoring determination for the meniscal extrusion^26^. Cartilage volume subregions include medial and lateral compartments (femoral condyle plus tibial plateau). Cartilage volume changes were calculated in % changes as the difference between time T + 1 and time T, divided by the cartilage volume at time T, and multiplied by 100.

The meniscal thickness acquired from the OAI Sagittal 3D DESS Water Excitation sequence underwent a multiplanar reformatting with the coronal plane well aligned with both posterior condyles. Three slices were selected: one in the coronal plane, allowing the thickness measurement of both meniscal bodies; and one for each meniscus in the sagittal plane for the measurement of the anterior and posterior horns. On the coronal plane, the selected slice was the first where the two condyles became separated when scanning the MR images from anterior to posterior (Supplementary Fig. S2A). The sagittal slices were those in which the entire meniscus shapes became triangular (Supplementary Fig. S2B). For both the medial and lateral menisci, the thickness of each of the three segments: anterior (sagittal) and posterior (sagittal) horns, as well as body (coronal), were measured using a MATLAB proprietary tool (MathWorks, Natick, MA, USA). Each assessment consisted of two measurements: the external thickness, with a measure at the outer edges of the meniscus where it joins the tibial plateau and the femoral condyle perpendicularly to the plateau; and the internal thickness, 6 mm inward from the external thickness measurement (Supplementary Fig. S2). The values of the mean thickness of the three segments of each meniscus were used as final values. An intra-reader validation was performed on 12 knees. Data reveal intra-class correlation (ICC) of 0.97 and 0.96 for the external and internal thickness of the medial meniscus, respectively, and an ICC of 0.98 and 0.97 for the lateral meniscus.

### Statistical analysis

Descriptive analyses of sociodemographic and clinical characteristics were conducted using proportion for categorical and mean (standard deviation [SD]) for continuous variables. Differences between groups were assessed using the Student’s t-test or Mann–Whitney test (non-normal distribution) for continuous variables, and the chi-squared test or Fisher’s exact test for categorical variables.

The changes in each treatment group over time were calculated for each follow-up period: T-1–T0, pre-treatment; T0–T1, treatment; T1–T2, post-treatment. The Skillings–Mack test (adapted for asymmetric distribution and missing data) was used to evaluate if the intragroup differences were statistically similar or different for both cases and controls.

To test whether there was any intergroup difference between cases and controls, analysis of covariance (ANCOVA) were done using different dependent variables (WOMAC pain, JSW, bone marrow lesions, bone curvature, mean meniscal thickness, cartilage volume) and independent variables comprising treatment, age, gender, BMI, meniscal extrusion, or, in the case of the cartilage volume change; cartilage volume, pain or arthritis medication and WOMAC pain. The intergroup comparison of synovial effusion volumes was tested using Student’s t-test for independent variables.

All tests were two-sided and a *p* value ≤ 0.050 was considered statistically significant. All statistical analyses were performed using SAS software, version 9.3 (SAS Institute, Cary, NC, USA).

## Results

From the OAI Incidence and Progression subcohorts, 89 participants (n = 93 knees) who fulfilled the inclusion criteria had received one injection of IACI and were matched to 93 knees (n = 90 participants) as controls (Fig. 1).

As the number of participants who had more than one injection fulfilling the inclusion criteria was very small (n = 10) and some had missing MRI exam(s) (n = 3) or JSW assessment(s) (n = 6), those participants were excluded because there was not enough information to draw any valid conclusions from a statistical analysis point of view.

### Participant characteristics

The two study groups (cases and controls) were balanced (Table 1) with the exception of a number of knees with KL grade 0 in the control group.

**Table 1:** Demographics, clinical and imaging characteristics.

	Intra-articular corticosteroid injection (n = 93*)	Control (n = 93*)	*p* value
**Index knee (right)—n (%)**	54 (58.1)	69 (74.2)	**0.020**^‡^
**Male—n (%)**	35 (37.6)	45 (48.4)	0.139^‡^
**Age (years)**	66.6 ± 9.7	66.2 ± 9.7	0.757^†^
	*(n* = *92)*		
**BMI (kg/m**^**2**^**)**	29.1 ± 4.1	28.1 ± 4.8	0.145^†^
**Height (cm)**	167.2 ± 8.4	168.8 ± 9.4	0.206^†^
**Symptoms duration—n (%)**	*(n* = *57)*	*(n* = *49)*	
< 5 years	25 (43.9)	22 (44.9)	0.915^‡^
> 5 years	32 (56.1)	27 (55.1)	
**WOMAC pain (score 0–20)**	4.3 ± 3.8	4.4 ± 3.9	0.952^†^
**Pain or arthritis medication at index date**—**n (%)**			
Acetaminophen	19 (20.4)	15 (16.1)	0.448^‡^
Narcotics	8 (8.6)	9 (9.7)	0.799^‡^
NSAIDs or COX-2 inhibitors	42 (45.2)	32 (34.4)	0.134^‡^
**Kellgren and Lawrence**—**n (%)**			
Grade 0	12 (12.9)	26 (28.0)	**0.004**^‡^
Grade 1	14 (15.1)	15 (16.1)	0.840^‡^
Grade 2	34 (36.6)	26 (28.0)	0.210^‡^
Grade 3	23 (24.7)	21 (22.6)	0.730^‡^
Grade 4	10 (10.8)	5 (5.4)	0.178^‡^
**Synovial effusion volume (mL)**	9.6 ± 8.3	8.6 ± 10.9	0.470^†^
	*(n* = *87)*	*(n* = *68)*	
**JSW (mm)**	3.7 ± 1.9	4.0 ± 1.64	0.511^†^
	*(n* = *20)*	*(n* = *14)*	
With meniscal extrusion	1.9 ± 1.6	2.1 ± 1.4	0.539^†^
	*(n* = *67)*	*(n* = *54)*	
Without meniscal extrusion	4.3 ± 1.6	4.5 ± 1.3	0.650^†^
**Cartilage volume (mm**^**3**^**)**			
Medial compartment	4,492.0 ± 1,364.0	4,957.9 ± 1616.0	0.064^†^
Lateral compartment	5,426.4 ± 1,334.0	5,598.7 ± 1,570.0	0.414^†^
**Meniscal Extrusion (Medial)—n (%)**	20 (21.5)	15 (16.1)	0.348^‡^
**BML (global knee)—n (%)**	43 (46.2)	40 (43.0)	0.658^‡^

Results are mean ± standard deviation (SD) or number (n) and percentage (%) of participants. *Number of knees studied.

The matching was done based on data at index time (T0) or at baseline (T-1).

BMI, body mass index; BML, bone marrow lesion; COX-2, cyclooxygenase-2; JSW, joint space width; NSAIDs, nonsteroidal anti-inflammatory drugs; WOMAC, Western Ontario and McMaster Universities Osteoarthritis Index.

All comparisons are made vs the control group, ^†^continuous variables were compared using Student's t-test/Mann–Whitney test; ^‡^proportions were compared using the Chi-squared test/Fisher’s exact test.

### Magnetic resonance imaging

The missing MRI data in the study groups were all related to missing MRI exams. More precisely, poor quality MRI exams that did not allow for the reliable assessment of the images (n = 2), lack of follow-up (n = 2), and the occurrence of total knee replacement (n = 22 knees) in the IACI group during the post-treatment period.

#### Cartilage volume change (Table 2)

For the medial compartment, the intragroup comparison showed that the control group had a smaller cartilage volume loss (*p* = 0.020) in the pre-treatment period compared to the treatment and post-treatment periods. For the lateral compartment, a numerical trend for smaller loss of cartilage volume (*p* = 0.052) was found in the post-treatment period in the IACI group.

**Table 2:** Cartilage volume change in knee compartments.

	Pre-treatment (T-1–T0)	Treatment (T0–T1)	Post-treatment (T1–T2)	*p* value*	(T0–T2)
**Medial compartment**					
Control	*(n* = *93)*	*(n* = *93)*	*(n* = *93)*		*(n* = *93)*
	− 0.33 ± 3.45	− 1.48 ± 4.29	− 1.95 ± 4.88	**(0.020)**	− 5.16 ± 8.38
IACI	*(n* = *91)*	*(n* = *93)*	*(n* = *67)*		*(n* = *67)*
	− 2.23 ± 4.02	− 1.72 ± 6.52	− 1.77 ± 5.18	(0.878)	− 3.53 ± 15.38
*p* value^†^	**(0.006)**	(0.676)	(0.638)		(0.883)
**Lateral compartment**					
Control	*(n* = *93)*	*(n* = *93)*	*(n* = *93)*		*(n* = *93)*
	− 0.52 ± 3.75	− 1.18 ± 3.81	− 0.69 ± 3.53	(0.159)	− 2.65 ± 5.82
IACI	*(n* = *91)*	*(n* = *93)*	*(n* = *67)*		*(n* = *67)*
	− 1.55 ± 3.76	− 2.37 ± 4.98	− 0.14 ± 3.90	(0.052)	− 3.64 ± 13.59
*p* value^†^	(0.071)	**(0.041)**	(0.678)		(0.247)

Results are mean ± standard deviation (SD) of cartilage volume change (%) and n, number of knees studied.

The change is computed as the difference of cartilage volume between time T+1 and time T.

*p* values in bold indicate statistical significance.

*Intragroup comparison was done using Skillings-Mack test.

^†^Intergroup comparison was done using an analysis of covariance (ANCOVA) where the dependent variable was the medial/lateral compartment cartilage volume at time T+1 and the independent variables were treatment (intra-articular corticosteroid injection [IACI] between T0 and T1), age, gender, body mass index, meniscal extrusion and medial/lateral compartment cartilage volume at time T.

T-1: Baseline, T0: month 12, T1: month 24, T2: month 36.

The intergroup comparison showed that the cartilage volume loss in the medial compartment in the pre-treatment period (T-1–T0) was significantly greater in the IACI group (*p* = 0.006), with a numerical trend (*p* = 0.071) in the lateral compartment. In the medial compartment during the treatment period (T0–T1) the cartilage volume loss was found to be similar in both groups, but a significantly greater cartilage loss was found in the lateral compartment in the IACI group (*p* = 0.041). However, in the post-treatment period (T1–T2) there was no difference in cartilage loss between the two groups in both compartments. In the two periods that included the treatment and post-treatment periods (T0–T2), no difference in cartilage loss was found between the groups for both compartments.

#### Bone curvature (Supplementary Table S1)

The differences in the change in bone curvature were in the lateral compartment where a significant intragroup difference was found in the control group (*p* = 0.007), showing a decrease in the loss of bone curvature in the treatment and post-treatment periods and a numerical trend in the IACI group (*p* = 0.072). Moreover, a significant difference was also noted between groups at the treatment period (T0-T1), where the IACI group showed a significantly smaller change than the control group (*p* = 0.037).

#### Bone marrow lesions (BML) (Supplementary Table S2)

The BML sizes were similar in control and IACI groups at baseline (T-1). Over time, the changes in BML were small and similar between control and IACI groups with no significant intra- or intergroup differences.

#### Synovial effusion (Supplementary Fig. S3)

The level of synovial effusion was similar in both treatment groups at T-1 with an increase over time which was more pronounced in the IACI group, more particularly following treatment.

#### Medial meniscal thickness (Table 3)

At baseline (T-1), the internal and external thickness of the medial (Table 3A) and lateral (Table 3B) meniscus were similar in both IACI and control groups. In the medial menisci, during the treatment period (T0–T1), a greater loss (*p* = 0.006) of the external thickness was found in the IACI group compared to the control group. This increase in loss of meniscus thickness in the IACI group was transient; the loss in the post-treatment period (T1–T2) was as in the pre-treatment period. For the period inclusive of treatment and post-treatment (T0–T2), a significantly greater cumulative thickness loss was recorded (*p* = 0.016) in the external segment of the medial meniscus, which corresponds to the increase in loss of thickness in the treatment period. For the lateral compartment (Table 3B), no differences in the internal or external meniscus thickness were found at any time between the two groups. The only exception was an intergroup difference in the control group (*p* = 0.044) for the external thickness which was small and unlikely to be of clinical significance.

**Table 3:** (A) Medial meniscal thickness and change. (B) Lateral meniscal thickness and change.

	T-1	Pre-treatment (T-1–T0)	Treatment (T0–T1)	Post-treatment (T1–T2)	*p* value*	(T0–T2)
**(A)**						
**Internal thickness**						
Control	*(n* = *85)*	*(n* = *85)*	*(n* = *85)*	*(n* = *85)*		*(n* = *85)*
	2.13 ± 0.62	− 0.03 ± 0.49	− 0.04 ± 0.51	− 0.03 ± 0.47	(0.237)	− 0.07 ± 0.60
IACI	*(n* = *80)*	*(n* = *80)*	*(n* = *82)*	*(n* = *61)*		*(n* = *61)*
	2.20 ± 0.87	− 0.13 ± 0.52	− 0.10 ± 0.51	− 0.14 ± 0.67	(0.800)	− 0.21 ± 0.77
*p* value^†^	(0.254)	(0.140)	(0.559)	(0.151)		(0.121)
**External thickness**						
Control	*(n* = *93)*	*(n* = *93)*	*(n* = *93)*	*(n* = *93)*		*(n* = *93)*
	5.37 ± 0.89	− 0.09 ± 0.45	− 0.01 ± 0.37	− 0.09 ± 0.48	(0.431)	− 0.10 ± 0.48
IACI	*(n* = *91)*	*(n* = *91)*	*(n* = *93)*	*(n* = *66)*		*(n* = *66)*
	5.32 ± 1.28	− 0.08 ± 0.60	− 0.20 ± 0.58	− 0.07 ± 0.56	(0.581)	− 0.30 ± 0.68
*p* value^†^	(0.926)	(0.721)	**(0.006)**	(0.790)		**(0.016)**
**(B)**						
**Internal thickness**						
Control	*(n* = *88)*	*(n* = *88)*	*(n* = *88)*	*(n* = *88)*		*(n* = *88)*
	1.92 ± 0.70	0.03 ± 0.33	− 0.04 ± 0.41	0.13 ± 0.84	(0.759)	0.09 ± 0.84
IACI	*(n* = *90)*	*(n* = *90)*	*(n* = *92)*	*(n* = *66)*		*(n* = *66)*
	2.03 ± 0.66	− 0.04 ± 0.47	− 0.05 ± 0.43	0.06 ± 0.47	(0.829)	0.05 ± 0.49
(*p* value)^†^	(0.107)	(0.236)	(0.729)	(0.474)		(0.547)
**External thickness**						
Control	*(n* = *93)*	*(n* = *93)*	*(n* = *93)*	*(n* = *93)*		*(n* = *93)*
	5.41 ± 1.05	0.02 ± 0.39	− 0.09 ± 0.37	− 0.05 ± 0.30	**(0.044)**	− 0.14 ± 0.37
IACI	*(n* = *90)*	*(n* = *90)*	*(n* = *92)*	*(n* = *66)*		*(n* = *66)*
	5.43 ± 0.96	− 0.04 ± 0.48	− 0.14 ± 0.63	− 0.05 ± 0.36	(0.142)	− 0.09 ± 0.58
(*p* value)^†^	(0.524)	(0.264)	(0.612)	(0.882)		(0.522)

Results are mean ± standard deviation (SD) of meniscal thickness (mm) or its change and n, number of knees studied.

The change is computed as the difference of meniscal thickness between time T+1 and time T.

*p* values in bold indicate statistical significance.

*Intragroup comparison of the changes in meniscal thickness was done using Skillings-Mack test.

^†^Intergroup comparison was done using an analysis of covariance (ANCOVA) where the dependent variable was the meniscal thickness or change in meniscal thickness at time T+1 and the independent variables were treatment (intra-articular corticosteroid injection [IACI] between T0 and T1), age, gender, body mass index, and meniscal extrusion.

T-1: Baseline, T0: month 12, T1: month 24, T2: month 36.

### X-ray (JSW) (Table 4)

The medial JSW was about similar in both groups at baseline (T-1). The loss in JSW in the pre-treatment period (T-1–T0) was slightly more pronounced in the IACI group, but the difference did not reach statistical significance. During the treatment period (T0–T1) the JSW loss was significantly greater (*p* = 0.011) in the IACI group. For the period inclusive of treatment and post-treatment (T0–T2), the patients in the IACI group had a significantly greater loss of JSW (*p* = 0.001).

**Table 4:** Medial joint space width (JSW) and change.

	T-1	Pre-treatment (T-1–T0)	Treatment (T0–T1)	Post-treatment (T1–T2)	*p* value*	(T0–T2)
Control	*(n* = *68)*	*(n* = *68)*	*(n* = *68)*	*(n* = *67)*		*(n* = *67)*
	4.06 ± 1.50	− 0.07 ± 0.47	− 0.17 ± 0.41	− 0.07 ± 0.62	(0.702)	− 0.23 ± 0.59
IACI	*(n* = *83)*	*(n* = *83)*	*(n* = *84)*	*(n* = *60)*		*(n* = *60)*
	3.87 ± 1.89	− 0.17 ± 0.58	− 0.48 ± 0.99	− 0.25 ± 0.55	(0.085)	− 0.78 ± 1.18
*p* value^†^	(0.591)	(0.243)	**(0.011)**	(0.077)		**(0.001)**

Results are mean ± standard deviation (SD) of joint space width (JSW) (mm) or its change and n, number of knees studied.

The change is computed as the difference of JSW between time T+1 and time T.

*p* values in bold indicate statistical significance.

*Intragroup comparisons of the changes in JSW were done using Skillings-Mack test.

^†^Intergroup comparisons were done using an analysis of covariance (ANCOVA) where the dependent variable was the medial JSW or its change at time T+1 and the independent variables were treatment (intra-articular corticosteroid injection [IACI] between T0 and T1), age, gender, body mass index, and meniscal extrusion.

T-1: Baseline, T0: month 12, T1: month 24, T2: month 36.

### Symptoms: WOMAC pain (Supplementary Table S3)

At baseline (T-1), the WOMAC pain scores (0–20, 20 worst) in both the IACI and control groups were low, indicating a mild level of pain in participants. For the intragroup comparison, in the pre-treatment period (T-1–T0) in both groups there was a slight increase in the WOMAC pain score change, indicating worsening pain. This was, however, followed by a decrease in the score change (less pain) in both groups at the follow-up periods. Those changes where small and, although they showed a statistical significance (control, *p* = 0.011) and a numerical trend (IACI, *p* = 0.054), were unlikely to be of clinical relevance. The intergroup comparison showed that at the time of the treatment (T0–T1) there was a lower level of pain in the control groups compared to the IACI group (*p* = 0.005).

## Discussion

This nested case–control study provides new and novel information on the safety of IACI on the evolution of knee structural changes in OA individuals, a topic of clinical interest in view of previous reports^10–14,27,28^ in which controversy still exists regarding possible deleterious effects on joint structure. Data in this study demonstrated that a single IACI in knee OA was safe with no negative impact on articular structural changes, as no differences between the control and IACI groups were found for cartilage volume change, bone curvature, and BML in the evolution of the structural changes post-treatment. However, data revealed a concomitant transient reduction of meniscal thickness and JSW, a finding for which the clinical relevance is at present unknown.

The study design, a nested case–control study, provides a new approach in the context of an observational cohort, such as the OAI, as it allows not only the exploration of the natural evolution of the disease, but the study and comparison of the potential role of therapeutic intervention on the evolution and outcome of the disease, while controlling for the most important relevant confounding factors. Such an approach has recently proven to be very informative when investigating the evolution of OA structural changes and the effects of treatment other than IACI on this disease progression and outcomes^19,20,29,30^.

For the first time, this study documents that IACI transiently reduce the thickness of the medial meniscus. The greater extent of thickness loss of the external portion of the medial meniscus occurred at the period surrounding the IACI. This finding correlates well with X-rays showing a decrease of JSW following the IACI and are in line with the known fact that the medial JSW could be influenced by the meniscal structure. For instance, the presence of meniscal extrusion has been found to be associated with a loss of JSW^31^. As the analysis was adjusted for meniscal extrusion, it is therefore most likely that the loss of JSW was truly related to the reduction in the meniscal thickness of the IACI participants. Possible explanations for the reduction in medial meniscal thickness are that the steroid injection could have a direct effect of reducing the local œdema level in the degenerated meniscus, which is a common phenomenon in OA, and/or reflects an anti-anabolic effect of the steroids on the meniscal matrix synthesis. Another possibility is that the steroid injection has an indirect effect and is related to a reduction of joint inflammation^32^.

This study ruled out the possibility that the difference in JSW reduction between the IACI and control groups is related to changes in bone structures such as BML or bone curvature, as no differences in changes between the two groups were found for these structures. One could also argue that the greater effusion size in the IACI group may have artefactually contributed to the extent of JSW reduction, which could have then been even more pronounced in that group. The exact cause for the increase in the size of effusion in the IACI group is unknown, but may reflect, at least in part, the volume introduced in the joint at the time of injection. However, an increase in the severity of synovitis seems unlikely as per previous reports^33,34^. The absence of effect of the IACI on the lateral meniscus is not very surprising as the structure of the meniscus in that compartment is usually better preserved than the medial one, with less matrix damage and œdema^16,26^.

The impact of the IACI in reducing meniscal thickness and JSW on disease outcome remains unknown, since there was no evidence of a negative impact of IACI on the cartilage volume in the period comprising treatment and post-treatment (T0-T2), and the period of meniscal thickness and JSW reduction was relatively short and mostly limited to the period following the injection.

Previous studies have reported no effects of IACI on JSW compared to control groups^11,13^. However, these studies were performed on OA patients with more severe disease (clinical trials) and no MRI assessment of joint structural changes was performed. A recent study by Zeng et al.^27^ using participants from the OAI cohort has concluded that IACI could induce a worsening of the JSW narrowing and total joint replacement. This study was also based solely on knee X-rays and again no MRI data were reported, therefore, the possibility that the JSW narrowing could be related to a reduction in meniscal thickness, instead of to a loss of cartilage, as reported in the present study, cannot be excluded. Moreover, in this aforementioned study^27^, important confounding risk factors of disease progression such as meniscal extrusion, BML, bone curvature and others were not included in the analysis, which makes it difficult to make firm conclusions about the association between IACI and worsening of the JSW. In addition, the fact that the propensity score matched participants were based on baseline variables and not on index times, as previously reported^19^, precludes any clear association between IACI and total knee replacement.

There was no deleterious effect on cartilage volume loss detected following the IACI over time. In fact, our results show that the rate of cartilage loss in the medial compartment in the IACI group, which was significantly greater than in the control group before treatment, was slightly reduced in the periods following the injection. Nevertheless, the level of cartilage volume loss remains within a range previously reported in knee OA^15–17^. The greater cartilage volume loss in the IACI group before the treatment likely reflects the greater severity of the disease found in these subjects, which could be the reason they received IACI. The higher level of cartilage loss in the IACI group in the lateral compartment at the treatment period (T0–T1) is intriguing as no difference was found between the groups over the T0–T2 period. The difference between the groups was small and the clinical relevance unknown at this time. Further investigation may be needed.

Data also showed no noticeable effect of IACI on disease symptoms (WOMAC pain, score 0–20) in post-treatment. This finding was not surprising since, as per the OAI database, the yearly visit did not allow for the timely estimation of the symptomatic effect of IACI over time, the effect of which usually lasts only a few weeks^8^. Moreover, the low level of pain at baseline may have also contributed to the findings.

Like any study, there were limitations. First, data were obtained from an observational study, therefore imposing restrictions on the study design and on the number of subjects that could be included in this study. In addition, in the OAI database, the fact that MRI were done every other year after the first five years also impacted the number of participants included in this study. Second, although the OAI database comprised a large amount of information that is very useful, there was no information available on the steroids’ preparation or dosage or exact time of administration, which could have provided more in-depth information on the structural effects of IACI. Third, based on the study design, the population of the present study consisted of individuals who received a single injection of IACI as only very few participants (n = 10) had received more than one IACI. Participants with repeated injections could therefore not be included in the study as several MRI and X-ray were missing for the first or subsequent injection, which precluded the conduct of valid comparative analysis. The fact that repeated IACI are not very frequent reflects a real-life scenario in which such treatment is used mostly at the time of disease flare up. Previous studies exploring the effect of repeated IACI on knee OA were done in a research context mainly to explore its potential as a disease-modifying OA drug, and do not seem to reflect common clinical practice.

In conclusion, this study showed that in OA, a single IACI was safe with no detrimental effects on the progression of knee structural changes, but with a transient reduction in medial meniscal thickness and JSW. The long-term clinical relevance of this finding remains, however, to be determined. All of the questions surrounding the safety of IACI in OA treatment will only find an answer in new, high-quality controlled clinical trials. Until then, “the jury is still out” on that very important and clinically relevant question.

## Supplementary information

Supplementary information

## Data Availability

Data used for this study were from the OAI database, which is publicly available online (https://nda.nih.gov/oai/). The data sets generated and/or analyzed during the current study are included in the published article or available from the corresponding author on reasonable request.
