# Supplementary material for: Intra-articular corticosteroid knee injection induces a reduction in meniscal thickness with no treatment effect on cartilage volume: a case–control study
Source: Sci Rep. 2020 Aug 14;10:13789. doi: 10.1038/s41598-020-70064-4 (PMC7427978; doi:10.1038/s41598-020-70064-4)
Supplement: Supplementary file 1 — Supplementary information [file 41598_2020_70064_MOESM1_ESM.docx]

**Intra-articular corticosteroid knee injection induces a reduction in meniscal thickness with no treatment effect on cartilage volume: A case-control study**

Jean-Pierre Pelletier^1*^, Jean-Pierre Raynauld^1^, François Abram^2^, Marc Dorais^3^,

Patrice Paiement^4^, Johanne Martel-Pelletier^1^

^1^ Osteoarthritis Research Unit, University of Montreal Hospital Research Centre (CRCHUM), Montreal, QC, H2X 0A9, Canada.

^2^ Medical Imaging Research & Development, ArthroLab Inc., Montreal, Quebec, Canada, H2K 1B6.

^3^ StatSciences Inc., Notre-Dame-de-l'Île-Perrot, Quebec, Canada, J7V 0S2.

^4^ ArthroLab Inc., Montreal, Quebec, Canada, H2K 1B6.

*Direct correspondance to: Jean-Pierre Pelletier, [dr@jppelletier.ca](mailto:dr@jppelletier.ca)

**SUPPLEMENTARY FIGURES AND TABLES**

**Figure S1. Study follow up visits**





M = Month

X represents intra-articular corticosteroid injection treatment time for a given patient.

Solid black vertical lines indicate follow-up visits from which data were collected: T-1, 0M; T0, 12M; T1, 24M; T2, 36M; T3, 48M.

**Figure S2**


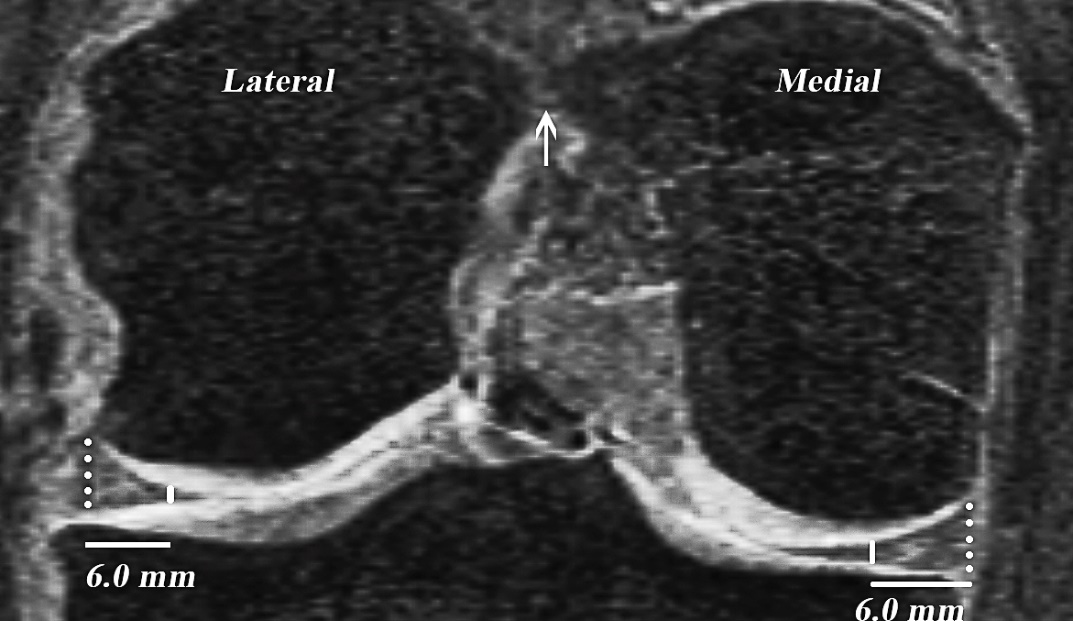


**Figure S2A**. Coronal view. Measurement of the external and internal thickness of the medial and lateral meniscal bodies. Arrow denotes the landmark position in the femur for the selection of the slice. Dotted line: External meniscal thickness; Solid line: Internal meniscal thickness.


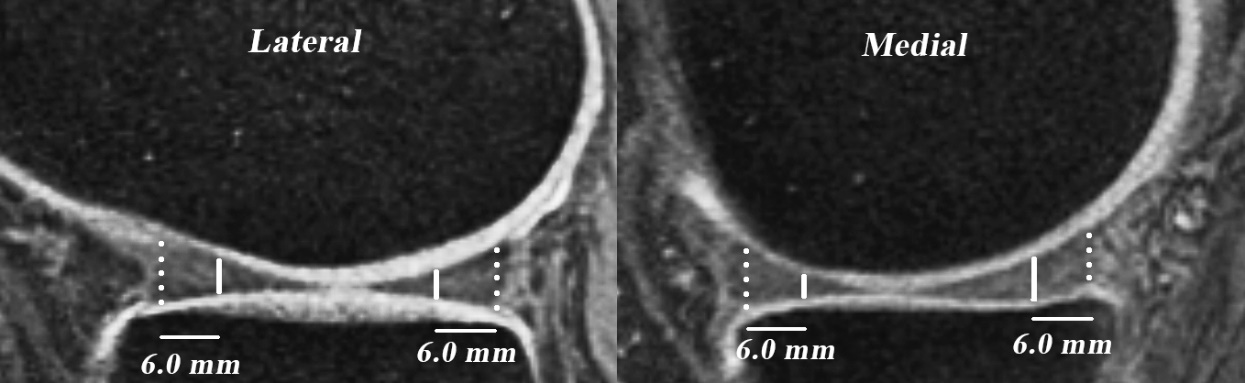


**Figure S2B.** Sagittal view. Measurement of the external and internal thickness of the meniscal horns (anterior and posterior). Arrows denote the landmark position in the meniscus for the selection of the slice on each laterality. Dotted line: External meniscal thickness; Solid line: Internal meniscal thickness.

**Figure S3. Synovial effusion volume (mL)**

**

**

Results mean ± standard deviation (SD) of synovial effusion volume (mL). Intra-articular corticosteroid injections (IACI) treatment between T0 and T1

T-1: Baseline (Control: n=93; IACI: n=93), T0: month 12 (Control: n=93; IACI: n=93), T1: month 24 (Control: n=93; IACI: n=93), T2: month 36 (Control: n=93; IACI: n=69).

† P-value for the intergroup comparison of synovial effusion volume (Student t-test).

**Table S1. Bone curvature change in knee compartments**

|  |  | **Pre-Treatment**  **(T-1 – T0)** | **Treatment**  **(T0 – T1)** | **Post-Treatment**  **(T1 – T2)** | **p-value*** | | **(T0 – T2)** |
| --- | --- | --- | --- | --- | --- | --- | --- |
| **Medial**  **Compartment** |  |  |  |  |  | |  |
|  |  | *(n=93)* | *(n=93)* | *(n=93)* |  | | *(n=93)* |
|  | **Control** | -0.84± 2.71 | -0.34 ± 3.48 | -0.50 ± 3.78 | (0.572) | | -0.85 ± 3.96 |
|  |  | *(n=88)* | *(n=90)* | *(n=66)* |  | | *(n=66)* |
|  | **IACI** | -0.75± 2.72 | -0.24 ± 2.01 | -0.75 ± 2.13 | (0.195) | | -0.85 ± 2.26 |
|  | **p-value†** | (0.821) | (0.919) | (0.800) |  |  | (0.963) |
| **Lateral**  **Compartment** |  |  |  |  |  | |  |
|  |  | *(n=93)* | *(n=93)* | *(n=93)* |  | | *(n=93)* |
|  | **Control** | -1.24 ± 2.26 | 0.14 ± 2.34 | -0.29 ± 3.14 | **(0.007)** | | -0.15 ± 2.87 |
|  |  | *(n=88)* | *(n=90)* | *(n=66)* |  | | *(n=66)* |
|  | **IACI** | -1.10 ± 3.05 | -0.89 ± 3.89 | 0.37 ± 4.21 | (0.072) | | -0.68 ± 3.33 |
|  | **p-value†** | (0.643) | (**0.037**) | (0.351) |  | | (0.367) |

Results are mean ± standard deviation (SD) of bone curvature change and n, number of knees.

The change is computed as the difference of bone curvature between time T+1 and time T.

*Intragroup comparison of the bone curvature changes was done using Skillings-Mack test.

†Intergroup comparison was done using an analysis of covariance (ANCOVA) where the dependent variable was the bone curvature change at time T+1 and the independent variables were treatment (intra-articular corticosteroid injection [IACI] between T0 and T1), age, gender, body mass index, and meniscal extrusion.

T-1: Baseline, T0: month 12, T1: month 24, T2: month 36.

**Table S2. Bone marrow lesions (BML) and change**

|  | **T-1**  (n=38) | **Pre-Treatment**  **(T-1 ‒ T0)**  (n=38) | **Treatment**  **(T0 ‒ T1)**  (n=38) | **Post-Treatment**  **(T1 ‒ T2)**  (n=38) | **p-value*** | | **(T0 ‒ T2)**  (n=38) |
| --- | --- | --- | --- | --- | --- | --- | --- |
|  |  |  |  |  |  | |  |
| **Control** | 3.60 ± 5.59 | -0.74 ± 5.92 | -0.08 ± 1.77 | 0.72 ± 5.27 | (0.918) | | 0.64 ± 5.36 |
|  |  |  |  |  |  | |  |
| **IACI** | 2.75 ± 1.58 | -0.08 ± 1.54 | 0.22 ± 2.41 | 0.09 ± 1.79 | (0.557) | | -0.21 ± 2.16 |
|  |  |  |  |  |  | |  |
| **p-value†** | (0.973) | (0.833) | (0.356) | (0.964) |  | | (0.316) |
|  |  |  |  |  |  |  |  |

Results are mean ± standard deviation (SD) of BML in the global knee (%) or its change.

The change is computed as the difference of BML in the global knee at time T+1 and time T.*Intragroup comparison of the changes in BML in the global knee was done using Skillings-Mack test.

†Intergroup comparison was done using an analysis of covariance (ANCOVA) where the dependent variable was the ln (natural logarithm) transformed BML or change in ln transformed BML at time T+1 and the independent variables were treatment (intra-articular corticosteroid injection [IACI] between T0 and T1), age, gender, body mass index, and meniscal extrusion.

T-1: Baseline, T0: month 12, T1: month 24, T2: month 36.

**Table S3. WOMAC pain score and absolute change**

|  | **T-1** | **Pre-Treatment**  **(T-1 ‒ T0)** | **Treatment**  **(T0 ‒ T1)** | **Post-Treatment**  **(T1 ‒ T2)** | **p-value*** | | **(T0 ‒ T2)** |
| --- | --- | --- | --- | --- | --- | --- | --- |
|  |  |  |  |  |  | |  |
|  | *(n=60)* | *(n=60)* | *(n=60)* | *(n=60)* |  | | *(n=60)* |
| **Control** | 4.38 ± 2.92 | 1.49 ± 3.15 | -1.28 ± 3.61 | -0.21 ± 3.27 | **(0.011)** | | -1.49 ± 3.59 |
|  |  |  |  |  |  | |  |
|  | *(n=70)* | *(n=70)* | *(n=70)* | *(n=70)* |  | | *(n=70)* |
| **IACI** | 4.36 ± 2.72 | 1.00 ± 3.84 | 0.51 ± 4.21 | -0.53 ± 4.00 | (0.054) | | -0.01 ± 3.77 |
|  |  |  |  |  |  | |  |
| **p-value†** | (0.647) | (0.160) | **(0.005)** | (0.201) |  |  | (0.079) |

Results are mean ± standard deviation (SD) of WOMAC pain score (scale 0-20, where 20 is worst) or change of WOMAC pain score.

The change is computed as the difference of WOMAC pain score between time T+1 and time T.

*Intragroup comparison of the changes in WOMAC pain score was done using Skillings-Mack test.

†Intergroup comparison was done using an analysis of covariance (ANCOVA) where the dependent variable was WOMAC pain score or change at time T+1 and the independent variables were treatment (intra-articular corticosteroid injection [IACI] between T0 and T1), age, gender, body mass index, and pain or arthritis medication.

WOMAC, Western Ontario and McMaster Universities Osteoarthritis Index

T-1: Baseline, T0: month 12, T1: month 24, T2: month 36.
